# Kupffer cells ameliorate hepatic insulin resistance induced by high-fat diet rich in monounsaturated fatty acids: the evidence for the involvement of alternatively activated macrophages

**DOI:** 10.1186/1743-7075-9-22

**Published:** 2012-03-22

**Authors:** Zuzana Papackova, Eliska Palenickova, Helena Dankova, Jana Zdychova, Vojtech Skop, Ludmila Kazdova, Monika Cahova

**Affiliations:** 1 Department of Metabolism and Diabetes, Institute for Clinical and Experimental Medicine, Videnska 1958/9, Prague 14021, Czech Republic; 2 Institute for Chemical Technology, Prague, Czech Republic

## Abstract

**Background:**

Resident macrophages (Kupffer cells, KCs) in the liver can undergo both pro- or anti-inflammatory activation pathway and exert either beneficiary or detrimental effects on liver metabolism. Until now, their role in the metabolically dysfunctional state of steatosis remains enigmatic. Aim of our study was to characterize the role of KCs in relation to the onset of hepatic insulin resistance induced by a high-fat (HF) diet rich in monounsaturated fatty acids.

**Methods:**

Male Wistar rats were fed either standard (SD) or high-fat (HF) diet for 4 weeks. Half of the animals were subjected to the acute GdCl_3 _treatment 24 and 72 hrs prior to the end of the experiment in order to induce the reduction of KCs population. We determined the effect of HF diet on activation status of liver macrophages and on the changes in hepatic insulin sensitivity and triacylglycerol metabolism imposed by acute KCs depletion by GdCl_3_.

**Results:**

We found that a HF diet rich in MUFA itself triggers an alternative but not the classical activation program in KCs. In a steatotic, but not in normal liver, a reduction of the KCs population was associated with a decrease of alternative activation and with a shift towards the expression of pro-inflammatory activation markers, with the increased autophagy, elevated lysosomal lipolysis, increased formation of DAG, PKCε activation and marked exacerbation of HF diet-induced hepatic insulin resistance.

**Conclusions:**

We propose that in the presence of a high MUFA content the population of alternatively activated resident liver macrophages may mediate beneficial effects on liver insulin sensitivity and alleviate the metabolic disturbances imposed by HF diet feeding and steatosis. Our data indicate that macrophage polarization towards an alternative state might be a useful strategy for treating type 2 diabetes.

## 

Obesity and type 2 diabetes have reached epidemic proportions in most of the Western world. Both conditions are strongly associated with non-alcoholic fatty liver disease (NAFLD) [1]. Obesity was first recognized as a chronic low-grade inflammatory condition of adipose tissue more than a decade ago [2]. Both liver and adipose tissue possess a specific macrophage subpopulation -"resident macrophages"- that undergo local activation in response to various stimuli and express distinct patterns of surface markers, chemokines and cytokines [3]. Depending on the triggering stimuli and genetic background, macrophages can undergo either a "classical" (Th1 dependent; M1) or "alternative" (Th2 dependent; M2) activation pathway. M1 response is an essential part of innate immunity and this pro-inflammatory program serves to protect the host against invading pathogens. However, if excessive, the inflammatory response becomes detrimental [4]. Many studies have supported the idea that inflammatory cytokine signaling directly promotes insulin resistance [5]. An "alternative" (M2) activation pathway results in a protective phenotype - it promotes maturation of alternatively activated macrophages to counteract excessive inflammation, enhance tissue repair and may have a beneficial role in regulating nutrient homeostasis [6].

Dietary fat intake has been long proposed as a causative factor for the development of metabolic syndrome. In this regard, not only the quantity, but mainly the quality of dietary fat consumed strongly predicts the prevalence of insulin resistance, type 2 diabetes and atherosclerosis [7,8]. Recent research suggests that most of the pro-inflammatory response of the innate immune system to invading pathogens can be traced to the unique family of membrane receptors known as TLRs [5]. Of great importance is the finding that TLRs (especially TLR2 and TLR4) recognize and are activated by the saturated fatty acyl moieties of bacterial lipopolysaccharide (LPS) while after substitution of saturated acyl moiety with unsaturated fatty acid the activity of LPS is lost [9,10]. The long experience, that diets enriched in SFA have been associated with increased risk for insulin resistance and type 2 diabetes [11] while dietary MUFA are protective against metabolic syndrome and cardiovascular disease risk factors [12,13] is perfectly in line with this finding.

An inflammatory response in the presence of obesity appears to be triggered by, and to reside predominantly in, adipose tissue [14]. In contrast to the adipose tissue, the accumulation of triacylglycerol droplets within hepatocytes (hepatic steatosis) is not generally associated with inflammation [15]. KCs, the resident liver macrophages, are the largest macrophage population in the body [16] and, in addition to fulfilling a variety of other immunologic functions [17,18], are the primary innate immune defense against exposure of foreign antigens from the diet and intestinal tract [18,19]. KCs are chronically exposed to higher concentrations of endotoxin than circulating peripheral blood monocytes. Therefore, it seems plausible that protective mechanisms have evolved to avoid the inadvertent activation of KCs while maintaining scavenger function [20]. In this connection, it is important to stress that resident hepatic macrophages display tremendous plasticity in their activation programs, ranging from the pro-inflammatory classical state to the anti-inflammatory alternative state [21,22]. While the rapid onset of inflammation in adipose tissue due to the HF diet feeding in adipose tissue is well established, there are only a few studies addressing the role of hepatic resident macrophages in the inflammatory and metabolically dysfunctional obese state and these studies have brought contradictory results. Some authors provide evidence that KCs may, at least partially, protect hepatocytes from the inflammatory milieu and the insulin resistance associated with a high-fat diet-induced obesity [6,23,24]. In contrast, other reports showed that hepatic macrophage response participates in the onset of high-fat diet-induced hepatic insulin resistance [25,26].

The aim of our study was to characterize the role of KCs in relation to the onset of hepatic insulin resistance induced by a HF diet rich in MUFA. In order to address this issue, we determined the effect of HF diet on the activation status of liver macrophages and the changes in hepatic insulin sensitivity and hepatic TAG metabolism imposed by acute KCs depletion by GdCl_3_, a specific KCs inhibitor, in standard and HF diet administered Wistar rats.

## Materials and methods

### Animals and experimental protocol

Male rats were kept in a temperature-controlled room at a 12:12-h light-dark cycle. Animals had free access to drinking water and diet if not stated otherwise. All experiments were performed in agreement with the Animal Protection Law of the Czech Republic 311/1997 which is in compliance with Principles of Laboratory Animal Care [27] (NIH Guide to the Care and Use of Laboratory Animals, 8^th ^edition, 2011) and were approved by the ethical committee of the Institute for Clinical and Experimental Medicine. Starting at age 3 months (b.wt. 300 20 g), animals were fed either HF diet (60 cal% as fat, 20 cal% as protein, 10 cal% as carbohydrate) or standard laboratory chow diet (SD) for 4 weeks. The FFA composition of the diet is given in Additional file 1. The groups designated as SD-starved or HF-starved were deprived of food for the last 24 hours, the groups labeled SD-fed or HF-fed had free access to the diet until decapitation (10 - 11 a.m.). When indicated, fed animals were administered insulin 30 min prior to decapitation (6 U/kg i.p.). Gadolinium chloride was administered in two doses 72 and 24 hrs prior to decapitation (10 mg/kg = 0.04 mM/kg i.p.).

### Oral glucose tolerance test

An oral glucose tolerance test (OGTT) was performed on a separate group of animals (n = 4). The rats were starved overnight and then given a single dose of glucose (3 g/kg b.wt.) *per os *dissolved in sterile saline. Blood was taken from the tail vein at 15, 30, 60, 120 and 180 min intervals and glucose was measured by Accu-check GO glucometer (Roche Diagnostics, Mannheim, Germany). Results were expressed as area under the curve (AUC).

### Real-time RT-PCR

The samples of liver tissue were dissected immediately after decapitation and frozen in liquid nitrogen. Total-RNA was extracted from tissue samples using Trizol reagent (Invitrogen) according to standard protocol [28]. A DNAase step was included to avoid possible DNA contamination. A standard amount of total RNA (1600 ng) was used to synthesize first-strand cDNA (High Capacity RNA-to-cDNA kit, Applied Biosystems, Foster City, CA). RT-PCR amplification mixtures (25 μl) contained 1 μl template cDNA, SYBER Green master mix buffer (Quanti-Tect, Qiagen, Hilden, Germany) and 400 nM (10 pmol/reaction) sense and antisense primer. Reactions were run on an Applera 7300 Real-Time PCR detector (Applied Biosystems). The results were analysed by SDS software version 2.3 (Applied Biosystems). The expression of genes of interest was normalised to the housekeeper gene (Ubc) and calculated using ΔΔCt method.

### Primer design

The primer sets were based upon known rat sequences available from the Rat Genome database http://www.rgd.mcw.edu. Primer design was performed with Primer3 software http://www.frodo.wi.mit.edu. Primer characteristics are listed in Additional file 2.

### Preparation of "light" and "dense" lysosomal fractions

The lysosomes and phagolysosomes represent a heterogeneous population of organelles. 20% (wt/vol) homogenate was prepared by homogenization of liver tissue in 0.25 M sucrose; 0.001 M EDTA pH = 7.4; heparin 7 IU/m, 1 mM PMSF, leupeptin 10 μg/ml, aprotinin 10 μg/ml by Teflon pestle homogenizer. The crude impurities were removed by brief centrifugation at 850 g. The fat cake was removed carefully in order to prevent contamination of liquid fraction. The homogenate was centrifuged for 10 000 g 20 min 4°C and the resulting pellet and supernatant were separated. The supernatant contains preferentially the less dense lysosomes with a higher TAG content ("light lysosomes"), the pellet is formed by more dense particles ("dense lysosomes").

### Assay of triacylglycerol lipase activity on exogenous substrate

The optimal conditions for the lipase assay (substrate concentration, reaction temperature and linear range of the assay) were determined as described previously [29]. Lysosomal subfractions prepared from the fresh tissue under iso-osmotic conditions were used for the assay. The reaction medium (92.5 kBq ^3^H triolein, 100 μM triolein, 110 μM lecithin, 0.15 M NaCl, 0.1 M acetate buffer pH = 4.5) was emulsified by sonication (Hielsler sonicator UP200S Teltow, Germany). The assay itself was performed under hypoosmotic conditions (50 mM sucrose) in order to ensure the release of the enzyme sequestered within the lysosomes. The liver homogenate or isolated fractions were incubated for 60 min at 30°C. The released fatty acids were extracted according to [30] and counted for radioactivity.

### Electrophoretic separation and immunodetection

Liver samples (200 mg) were harvested in situ and stored in liquid nitrogen until further utilization. The homogenate was prepared by Ultra-Turax homogenizer (IKA Worke, Staufen, Germany) in a homogenization buffer (150 mM NaCl, 2 mM EDTA, 50 mM TRIS, 20 mM glycerolphosphate, 1 mM Na_3_VO_4_, 2 mM sodium pyrophosphate, 1 mM PMSF, leupeptin 10 μg/ml, aprotinin 10 μg/ml). The proteins were separated by electrophoretic separation under denaturating conditions and electroblotted to PVDF membranes. The level of phosphorylation of PKB kinase and insulin receptor (IR) was assessed by immunodetection using specific antibodies. The total expression of PKB and IR protein was determined on the same membrane after striping and reblotting using specific antibodies. LC3-II content was determined in the 20% liver homogenate lysed by 2% SDS at 100°C. The loading control was performed using rabbit polyclonal antibody to beta actin. The list of antibodies employed in this study is given in Additional file 3. The bands were visualized using ECL and quantified using FUJI LAS-3000 imager (FUJI FILM, Tokyo, Japan) and Quantity One software (Biorad, Hercules, CA).

### Determination of autophagy intensity

The most frequently used autophagy marker is the quantification of microtubule-associated protein 1 light chain 3 (LC3). LC3 is initially synthesized in an unprocessed form, proLC3, which is converted into proteolytically processed form lacking amino acids from the C-terminus, LC3-I, and is finally modified into a phosphatidylethanolamine-conjugated form, LC3-II. LC3-II is the only protein marker that is reliably associated with phagophores, sealed autophagosomes and mature autophagosomes/autolysosomes [31]. The LC3-I and LC3-II content was determined as described above and the autophagy intensity was estimated as the LC3-II:LC3-I ratio.

### Determination of DAG content

This method is based on the phosphorylation of DAG in the sample to DAG-3-phosphate using γ^35^-ATP followed by quantification of radioactivity in a chlorophorm extract. Lipids from liver tissue or incubation mixture were extracted in chloroform/methanol and an aliquot of chlorophorm phase was evaporated under a stream of nitrogen. The sample was then solubilised by sonication in detergent buffer (7.5% n-octyl-β-D-glucopyranoside, 5 mM cardiolipin,1 mM DETAPAC). Reaction buffer (50 mM imidazol/HCl, pH = 6.6, 50 mM NaCl, 12.5 mM MgCl_2_, 1 mM EGTA), diacylglycerol kinase and γ^35^-ATP were added and incubated 30 min in 25°C. Lipids were extracted into chloroform/methanol, phases were separated with 1% HClO_4 _and the exact volume of lower chlorophorm phase was determined. An aliquot was evaporated, resolved in 5% chloroform/methanol and separated by TLC. Individual populations of lipids were visualised by iodine vapours, the bands corresponding to DAG were scraped off and the radioactivity was determined by scintillation counting.

### Biochemical analyses

TAG content in liver homogenate was determined after the extraction according to Folch [32]. The glycogen content was determined in homogenate after hydrolysis in 30% KOH and expressed as a glucose equivalent (μmoles per g wet weight). Protein concentration, FFA, - hydroxybutyrare, insulin and TAG serum content were determined using commercially available kits (protein concentration: QuantiPro BCA Assay kit, Sigma-Aldrich, St. Louis, MO; FFA: FFA half micro test, Roche Diagnostics, Mannheim Germany; triglycerides: Pliva-Lachema, Brno Czech Republic; insulin: Mercodia, Uppsala Sweden; hydroxybutyrate: RanBut, RANDOX, Crumlin, UK).

### Statistical analysis

Data are presented as mean SEM. Statistical analysis was performed using Kruskal-Wallis test with multiple comparisons (n = 5 -7). Differences were considered statistically significant at the level of p < 0.05.

## Results

### Effect of Kupffer cells reduction on lipid and glucose metabolism

At the end of the experiment, we found no difference in body weight between experimental groups (table 1). As expected, the adiposity (expressed as the percent of epididymal fat pad weight of the total body weight) was higher in HF diet administered group but it was not affected by the gadolinium treatment. HF diet administration significantly affected FFA metabolism and abolished the prandial dependent variation in serum FFA concentrations. Acute gadolinium treatment (two doses 24 and 72 hrs prior the experiment) had no effect on weight gain and adiposity (Additional file 4: Table S1). This resulted in a significant elevation of fed FFA serum levels in both SD and HF groups. Concentration of ketone bodies in serum may serve as an indicator of FFA metabolism in the liver. The fasting concentration of ketone bodies in serum was similar in all groups. Both HF diet administration and gadolinium treatment independently led to the increased ketogenesis in fed state and their effect was additive. HF diet administered animals exhibited higher triglyceridemia when compared to their SD fed littermates. GdCl_3 _treatment led to a substantial decrease of plasma TAG in HF group while having no effect in SD group. Fasting serum glucose was comparable in all groups. Fasting insulinemia was moderately elevated due to the HF diet feeding and GdCl_3 _administration significantly exacerbated this trend. As expected, whole body glucose tolerance determined as area under the curve (AUC) during the oral glucose tolerance test was worsened in HF group compared with SD fed animals. In the GdCl_3 _treated HF group, we observed a trend to the worsening of glucose tolerance but it did not reached statistical significance (p = 0.063). Nevertheless, a significant difference was found in the course of OGTT curve, the glucose concentration in 60 min being significantly higher in HF + GdCl_3 _when compared with HF diet-only fed animals. No effects were observed in SD group. In conclusion, KCs reduction influenced selected parameters of lipid and glucose metabolism but only in HF group.

**Table 1:** Effect of HF diet and Kupffer cells reduction on lipid and glucose metabolism parameters in serum

		SD	SD + GdCl_3_	*P*	HF	HF + GdCl_3_	*P*
body weight (g)		359 ± 3.4	369 ± 11.2	*N.S*.	365 ± 6.2	372 ± 8.5	*N.S*.

adiposity (%)		1.2 ± 0.03	1.4 ± 0.07	*N.S*.	1.6 ± 0.18^x^	1.8 ± 0.06	*N.S*.

s-FFA (mmol/l)	starved	0.8 ± 0.1	0.81 ± 0.04	*N.S*.	0.54 ± 0.04^x^	0.46 ± 0.01^y^	*N.S*.
	
	fed	0.27 ± 0.02	0.43 ± 0.02	*0.01*	0.5 ± 0.03^x^	0.67 ± 0.02^y^	*0.01*

s-ketone bodies (mmol/l)	straved	3.1 ± 0.1	3.2 ± 0.1	*N.S*.	3.1 ± 0.2	2.8 ± 0.4	*N.S*.
	
	fed	0.16 ± 0.02	0.34 ± 0.01	*0.01*	0.5 ± 0.05^x^	1.2 ± 0.2^y^	*0.001*

s-TAG (mmol/l)	fed	1.1 ± 0.1	1.0 ± 0.1	*N.S*.	2.0 ± 0.3^x^	0.8 ± 0.1	*0.001*

s-glucose (mmol/l)	starved	4.3 ± 0.2	4.7 ± 0.3	*N.S*.	4.6 ± 0.3	4.6 ± 0.2	*N.S*.

s-insulin (ng/l)	starved	112 ± 6	125 ± 8	*N.S*.	126 ± 10^x^	161 ± 8^y^	*0.05*
	
	fed	544 ± 29	474 ± 37	*N.S*.	472 ± 18	467 ± 12	*N.S*.

OGTT AUC_180 min_		1168 ± 27	1094 ± 41	*N.S*.	1305 ± 29^x^	1370 ± 15^y^	*0.063*

OGTT glucose 60 min, mM		6.5 ± 0.2	6.4 ± 0.24	*N.S*.	8.7 ± 0.3^x^	9.5 ± 0.21^y^	*0.05*

Fasted animals were deprived of food for 24 hours, fed animals had free access to food. Adiposity was expressed as the ratio of epididymal fat pad weight to the total body weight [(EWAT/b.wt.)*100]. OGTT = oral glucose tolerance test; AUC = cumulative area under the curve for blood glucose over 180 min. *P value *indicates the significance of GdCl_3 _treatment within each group; ^x ^p < 0.05 HF vs SD; ^y ^p < 0.05 HF + GdCl_3 _vs SD + GdCl_3_. Data are given as mean ± S.E.M., n = 6; OGTT n = 4.

### Kupffer cells reduction increases lysosomal degradation of TAG

As it has been recently shown in hepatocytes, a significant portion of TAG is degraded in lysosomes in the liver and the autophagy pathway has been proposed as one of the mechanisms ensuring the TAG degradation in hepatocytes [33]. According to this hypothesis, the lipid droplets are engulfed by autophagolysosomal membrane and transported via autophagy mechanisms into the lysosomes for degradation. As it was previously demonstrated [29], these activated lysosomes are less dense ("light") and could be separated from the inactive lysosomal pool ("dense") by differential centrifugation [34]. Lysosomal lipase (LAL) is the only lysosomal lipase and this enzyme is active solely within the lysosomes. It seems that the critical point in determining its physiological activity is the transportation of the substrate (lipid droplets) to the site of degradation (lysosomes). Based on these assumptions, we tested the effect of KCs reduction on lysosomal TAG degradation according to three parameters: 1. the distribution of LAL activity among the light and dense lysosomes; 2. the distribution of LAL protein among the light and dense lysosomes and 3. the autophagy intensity determined as LC3-II/LC3-I ratio.

**Figure 1:**
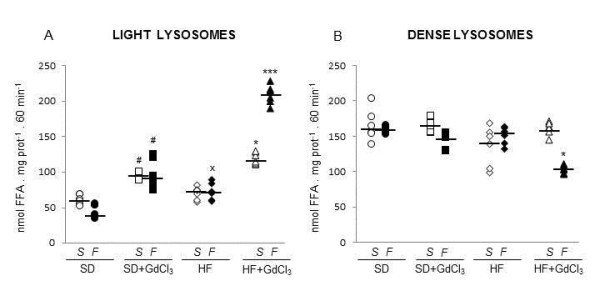
**Effect of HFdiet and Kupffer cells reduction on lysosomal lipase activity measured as FFA release from artificial substrate (^3^H-triolein)**. A: light lysosomes; B: dense lysosomes. The lipase activity was measured as the release of fatty acids at pH = 4.5 from^3^H triolein. All data are presented together with median. Open symbols = starved animals (S); closed symbols = fed animals (F). ○ ● SD; □ ■ SD + GdCl_3; _♦ ◊ HF; ▲ Δ HF + GdCl_3_. ^# ^p < 0.05 SD + GdCl_3_vs SD; *p < 0.05, *** p < 0.001 HF + GdCl_3 _vs HF; ^x^p < 0.05 HF vs SD.

**Figure 2:**
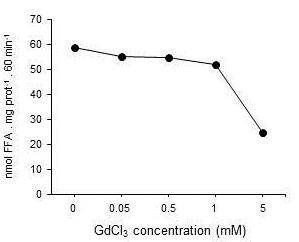
**Effect of GdCl_3 _on lysosomal lipase activity in vitro**. The GdCl_3 _was added to the sample 15 min prior the start of the assay; the lipase activity was measured as the release of fatty acids at pH = 4.5 from ^3^H triolein. Data represent an average from three independent experiments.

As shown on Figure 1A, fasting LAL activity associated with light lysosomal fraction (i.e. physiologically active enzyme) was not influenced by diet. In fed animals, LAL activity in HF group was elevated by approx. 50% compared to their littermates administered SD. KCs reduction had a significant stimulatory effect on LAL activity associated with light lysosomal fraction in both dietary groups and both in fed and starved animals. Nevertheless, while in SD-starved, SD-fed and HF -starved groups this elevation remained in the range of approx 50%, while this elevation in HF -fed group reached more than 200%. In contrast to light lysosomal fraction, the LAL activity determined in dense lysosomes was comparable in all groups with the exception of the GdCl_3_-treated HF -fed animals where we had observed an approximate 45% decrease of the enzyme activity (Figure 1B). As shown in Figure 2, GdCl_3 _in lower doses (< 1 mM) had no direct effect on LAL activity in vitro and in higher doses it even inhibited it. This finding indicates that the observed effect should be ascribed to the KCs reduction and not to the artificial stimulation of the enzyme.

**Figure 3:**
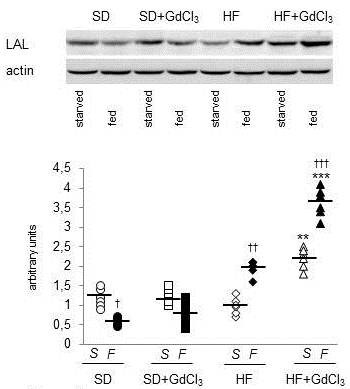
**Effect of HF diet and Kupffer cells reduction on lysosomal lipase protein abundance in light lysosomal fraction**. Lysosomal lipase can degrade intracellular TAG only when lipid droplets and LAL co-localize in autophagolysosomes. Autophagolysosomes "light" lysosomes) are less dense than inactive lysosomes due to their content of less denselipid droplets and could be separated by differential centrifugation. The light lysosomal fraction was prepared as described in Material and Methods. All data are presented together with median. Open symbols = starved animals (S); closed symbols = fed animals (F). ○ ● SD; □ ■ SD + GdCl_3_; ♦ ◊ HF;▲ Δ HF + GdCl_3_.^†^p < 0.05,^† †^p < 0.01,^† † †^p < 0.001 fed vs starved; ** p < 0.01, *** p < 0.001 HF + GdCl_3 _vs HF.

**Figure 4:**
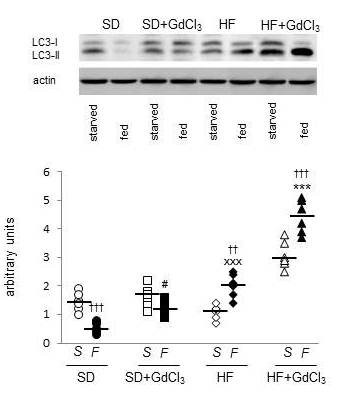
**Effect of HF diet and Kupffer cells reduction on autophagy intensity determined as LC3-II expression in the liver**. During the autophagosome formation, cytosolic LC3-I protein is lipidated to form LC3-II. LC3-II is incorporated into autophagosome membrane and remains there during the whole autophagy process until the stage of late lysosome. The formation of LC3-II is thus considered to be an indicator of autophagy intensity. The quantification of LC3-I and LC3-II in liver homogenate was performed by immunodetection. Data are expressed as LC3-II/LC3-I ratio. All data are presented together with median. Open symbols = starved animals (S); closed symbols = fed animals (F). ○ ● SD; □ ■ SD + GdCl_3_; ♦ ◊ HF;▲ Δ HF + GdCl_3_. ^† †^p < 0.01, ^† † †^p < 0.001 fed vs starved;^#^p < 0.5 SD + GdCl_3 _vs SD; *** p < 0.001 HF + GdCl_3 _vs HF; ^xxx^p < 0.001 HF vs SD.

The abundance of LAL protein in the light lysosomal fraction followed the same trend as the distribution of LAL activity. As shown on Figure 3, HF diet itself resulted in the increased LAL protein content in light lysosomes of the fed animals. The effect of HF diet was markedly exacerbated by GdCl_3 _treatment. Similar results were obtained when we determined the autophagy intensity as the LC3-II/LC3-I ratio in the liver homogenate (Figure 4). As expected, we observed a significant decrease of autophagy intensity in SD-fed compared with the SD-starved animals. HF diet abolished this regulation by increasing the autophagy intensity especially in the fed state and this effect was significantly accentuated in fed animals of HF + GdCl_3 _group. This data shows that the reduction of KCs population is associated with the increased lysosomal lipolysis of liver TAG and suggests the possibility that an increased autophagy may underlay the observed pro-lypolitic effect.

### Kupffer cells reduction increases the formation of TAG metabolism intermediates in the liver

KCs reduction was associated with lower TAG content in the liver of the HF group (table 2) and with increased ketogenesis (table 1) which provides further indirect evidence supporting the stimulatory effect of GdCl_3 _treatment on TAG liver degradation. The increased TAG breakdown may be associated with the increased formation of TAG degradation products. As shown in table 2, GdCl_3 _treatment significantly elevated DAG content in HF group exacerbating the effect imposed by HF diet itself. No changes in liver TAG and DAG content due to the GdCl_3 _administration were observed in the SD group. Taken together, our data suggest that in steatotic liver KCs reduction leads to the increased production of potentially hazardous lipid metabolism intermediates like DAG.

**Table 2:** Effect of HF diet and Kupffer cells reduction on triacylglycerol content in the liver

		SD	SD + GdCl_3_	*P*	HF	HF + GdCl_3_	*P*
TAG (μmol/g)	fasted	7.1 ± 0.2	8.1 ± 0.4	*N.S*.	21.4 ± 1.4^x^	14.1 ± 1.7^y^	*0.05*
	
	fed	4.4 ± 0.4	5.3 ± 0.4	*N.S*.	25.2 ± 0.7^x^	16.4 ± 0.3^y^	*0.01*

DAG (nmol/g)	fasted	10.8 ± 0.3	12.4 ± 0.6	*N.S*.	31.2 ± 1.1^x^	54.6 ± 2.5^y^	*0.01*
	
	fed	7.3 ± 0.2	6.8 ± 0.2	*N.S*.	36.7 ± 1.8^x^	69.3 ± 2.1^y^	*0.001*

*P value *indicates the significance of GdCl_3 _treatment within each group; ^x ^p < 0.05 HF vs SD; ^y ^p < 0.05 HF + GdCl_3 _vs SD + GdCl_3. _Data are given as mean ± S.E.M., n = 6.

### Kupffer cells reduction exacerbates the manifestation of HF diet induced hepatic insulin resistance

**Figure 5:**
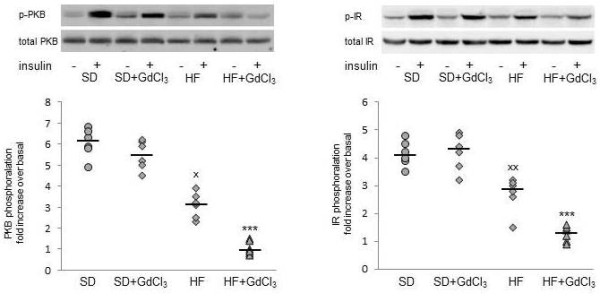
**Effect of HF diet and Kupffer cells reduction on insulin signalling**. A: PKB (Ser473) phosphorylation, B: insulin receptor (phospho Y1158) phosphorylation. The results are expressed as fold increase in the insulin-stimulated state relative to the basal state. Representative Western blot is shown above each graph. The basal level of PKB-and IR-phosphorylation was determined in the homogenate prepared from the liver of 24 hours starved animals. The effect of insulin was determined in identically processed samples from animals which had free access to food and 30 min prior to decapitation were administered insulin 6 U/kg. The total PKB and IR expression was determined after striping the membranes and re-blotting with anti-PKB and anti-IR antibodies, resp. All data are presented together with median. gray ○ SD; gray □ SD + GdCl_3_; gray ◊ HF; gray Δ HF + GdCl_3_. *** p < 0.001 HF + GdCl_3 _vs HF; ^x ^p < 0.05, ^xx ^p < 0.01 HF vs SD.

HF diet administration is associated with rapid onset of hepatic insulin resistance [35]. In our experiments, four weeks of HF diet administration resulted in attenuation of insulin-stimulated PKB and insulin receptor phosphorylation (Figure 5) and in the significant diminution of insulin-stimulated liver glycogenesis (table 3). Ablation of KCs had no effect on hepatic insulin sensitivity in the SD group but it severely exacerbated insulin resistance in the liver in HF diet administered animals. We detected virtually no effect of insulin on PKB phosphorylation and only very mild insulin-stimulated phosphorylation of insulin receptor on tyrosine. The insulin-stimulated glycogenesis was decreased compared with the untreated HF group. These results indicate that KCs depletion is associated with the further deterioration of hepatic insulin sensitivity already provoked by HF diet.

**Table 3:** Effect of HF diet and Kupffer cells reduction on glycogen content in the liver

		SD	SD + GdCl_3_	*P*	HF	HF + GdCl_3_	*P*
glycogen (μmol/g)	starved	12 ± 2.6	65 ± 8.5	*0.01*	17 ± 1.6	49 ± 8	*0.05*
	
	fed + ins	226 ± 13	196 ± 13	*N.S*.	198 ± 19	110 ± 18	*0.01*

Animals in "fed + ins" group were provided food *ad libitum *and 30 min prior decapitation were administered insulin (6 U/kg b.wt.) i.p. The glycogen is expressed in glucose equivalents (μmoles per g wet weight) *P value *indicates the significance of GdCl_3 _treatment within each group; ^y ^p < 0.05 HF + GdCl_3 _vs SD + GdCl_3_. Data are given as mean ± S.E.M., n = 6.

### Kupffer cells reduction combination with HF diet is associated with the increase in PKCε activity

**Figure 6:**
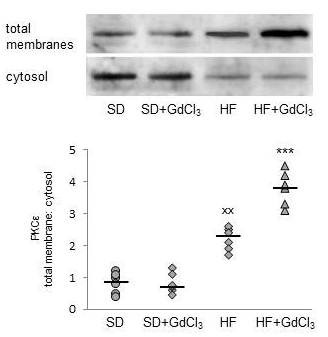
**The effect of HF diet and Kupffer cells reduction on PKCε activation in the liver**. Representative Western blot is shown in the upper part of the figure. The PKCε activation was assessed according to its translocation from cytosolic to membrane fraction and expressed as the ratio of relative densities of the bands in the membrane fraction and the corresponding ones in cytosolic fraction. All data are presented together with median. gray ○ SD; gray □ SD + GdCl_3_; gray ◊ HF; gray Δ HF + GdCl_3_. ^xx^p < 0.01 SD vs HF; *** p < 0.001 HF + GdCl_3 _vs HF.

PKCε has been implicated as a key player in the onset of HF diet-induced hepatic insulin resistance. PKCε activation is reflected by its translocation from cytosol to the plasma membrane and we used the ratio of relative abundance of PKCε in the membrane and cytosol fractions as the parameter indicating PKCε activity. As shown in Figure 6, in animals fed SD PKCε total membrane/cytosol ratio is not influenced by GdCl3 administration. HF diet itself led to the increased translocation of PKCε to the total membrane fraction. Reduction of KCs population was associated with a marked increase of PKCε abundance in total membranes at the expense of its content in the cytosol.

### HF diet does not lead to the expansion of hepatic Kupffer cell population but increases the expression of alternative activation pathway markers

**Figure 7:**
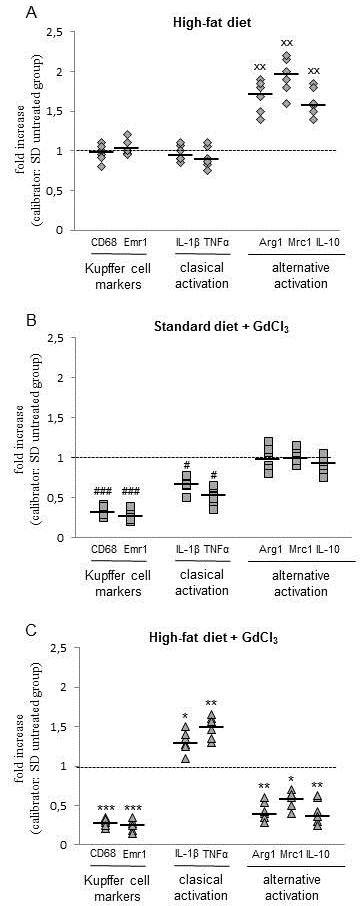
**Effect of HF diet and Kupffer cells reduction on macrophage classical and alternative activation markers**. A: HF vs SD group; B: SD + GdCl_3 _vs SD group; C: HF + GdCl_3 _vs SD group. GdCl_3 _was administered in two doses 72 and 24 hrs prior the experiment (10 mg/kg, i.p.). The expression of individual macrophage markers mRNA was determined by RT-PCR. Data are expressed as fold change related to untreated SD group with expression in standard diet (SD) arbitrary set at 1 (dotted line). gray ○ SD; gray □ SD + GdCl_3_; gray ◊ HF; gray Δ HF + GdCl_3_. ^xx^p < 0.01 SD vs HF; ^#^p < 0.05, ^### ^p < 0.001 SD + GdCl_3 _vs SD; *p < 0.05, **p < 0.01, ***p < 0.001 HF + GdCl_3 _vs HF.

The levels of two macrophage markers, CD68 and Emr-1 (F4/80), were used to assess the size of the resident macrophage population in the liver. The expression of either of these two markers remained unaltered due to the HF diet feeding compared with SD group. The expression of TNFα, classical (pro-inflammatory) activation pathway marker, was not affected in the HF group. In contrast to this, the alternative pathway was significantly activated in the liver of animals fed HF diet as demonstrated by the increase of the expression of Arg-1, Mrc-1 and IL-10 (Figure 7A). Gadolinium administration led to the approx 60% and 70% reduction of CD 68 and Emr-1 expression, resp., and this reduction was not dependent on the diet (Figure 7B and 7C). In the SD group, the expression of classical pro-inflammatory activation markers was decreased while the alternative activation was not significantly influenced by this treatment. In contrast, in the HF group, GdCl_3 _treatment resulted in a significant increase of the expression of IL-1β and TNFα and to a significant decrease of the expression of Arg-1, Mrc-1 and IL-10 mRNA. Taken together, our data reveals that administration of HF diet rich in MUFA itself results in alternative activation of liver macrophages. Furthermore, the reduction of the KCs population by gadolinium treatment resulted in a drastic reduction of alternatively activated population of liver macrophages and to a significant up-regulation of the expression of classical, i.e. pro-inflammatory markers in steatotic liver. No such effect was observed in the SD group.

## Discussion

In the present study, we provide evidence that KCs under certain circumstances play a protective role in the development of steatosis-induced insulin resistance. Our conclusion is based on several findings. A HF diet rich in MUFA is associated with a shift towards alternative rather than classical activation pathway of macrophages. In the steatotic liver, the reduction of KCs population by GdCl_3 _negatively affected alternatively activated macrophages and enhanced the expression of pro-inflammatory markers TNFα and IL-1β It was further associated with a significant increase of autophagy, lysosomal lipolysis, increased production of potentially hazardous TAG metabolism intermediates (DAG) and PKCε activation in the liver. Finally, the KCs reduction resulted in a significant exacerbation of HF diet-induced hepatic insulin resistance.

It has been recognized that KCs fulfill a dual role - they may function either as mediator of damage or as a protector during processes of regeneration and repair [36]. Strategies employing depletion or modulation of KCs, such as application of gadolinium chloride or liposome-encapsulated dichloromethylene bisphosphonate (Cl2-MBP) were effective to protect against liver injury induced by thioacetamide [37], carbon tetrachloride [38], alcohol [39] and ischemia/reperfusion [40]. On the other hand, there are clear indications that KCs can mediate protection and that their depletion increases liver injury after hepatectomy [41] or after total hepatic ischemia/reperfusion injury with bowel congestion [42].

Nevertheless, the role of KCs in the modification of metabolic fitness of the liver remains enigmatic. Several recent studies explored the role of KCs in relation to hepatic steatosis and insulin resistance which reported conflicting results since KCs depletion has been associated both with improvement [23] or worsening [43] of hepatic insulin resistance. Unfortunately, the described experiments vary greatly in experimental design which may profoundly influence the conclusions drawn from these studies. We can identify several "areas of disagreement" in this field.

In contrast to adipose tissue, the literature is not uniform about the effect of HF diet on the macrophage infiltration into liver. Some studies [25,44,45] report a slight increase in liver macrophages but most of the authors found no macrophage infiltration in response to HF diet [23,46-48]. In our study, we found no changes either in CD68 or Emr1 expression in HF diet administered animals compared with the SD group. Similarly, quite opposite results were reported when the activation status of liver macrophages was concerned. Classical (i.e. proinflammatory) activation was reported after 2 weeks of HF diet based on milk fat [43] containing approx. 70% of SFA and 1-5% of trans FA, or after 3 days of HF diet containing lard [25]. The later data are derived from a 3-day diet administration which indicates the existence of early inflammatory phase that occurs during the first days of HF diet administration and may contribute to the rapid onset of hepatic insulin resistance. Nevertheless, with the increasing duration (15 wks) of feeding HF diet containing MUFA (lard), the alternative activation of KCs prevails and imposes the immune-tolerant, anti-inflammatory state of the liver [16]. This could be considered as an adaptive mechanism counteracting the negative influence of a HF diet. Recent evidence shows that an important factor driving the switch between pro- and anti-inflammatory programs of resident macrophages may be the composition of dietary fat and, depending on the fatty acid composition, the effect may be highly variable; the pro-inflammatory potential is attributed to SFA while MUFA and PUFA exhibit an inhibitory effect on Toll like receptors-dependent inflammatory pathway and rather stimulate alternative activation. Shi et al. [47] demonstrated that SFA (particularly C14:0, C16:0 and C18:0) stimulated TNFα and IL-6 production from RAW246.7 macrophage cell line while no stimulation was observed after treatment with MUFA or PUFA fatty acids. Pretreatment of RAW 264.7 macrophages with palmitate resulted in their pro-inflammatory activation and the conditioned palmitate-free medium from these macrophages which was capable of inducing insulin resistance in myoblasts [49,50]. Wen et al. [51] reported that saturated fatty acid (palmitate) but not unsaturated fatty acid (oleate) induced NLRP3-ASP inflammasome activation in hematopoietic cells. In contrast, PUFA (especially EPA and DHA) treatment ameliorated LPS-induced pro-inflammatory signaling in macrophages and enhanced the insulin sensitivity of 3T3L1 adipocytes in co-culture experiments [52,53]. We have previously shown that 4 wks administration of the diet rich in *trans *FA (60 cal% fat; 50% of fat in the form of *trans *MUFA) resulted in a highly significant elevation of TNFα and IL-1β expression in the liver [54].

In our experiments, quantitative RT-PCR revealed that 4 weeks of administration of the HF diet rich in MUFA led to the increased expression of arginase-1, manose receptor and IL-10 which indicates the alternative activation of liver macrophages. In contrast to this, we did not find any sign of the increased pro-inflammatory signaling. Interestingly, in HF group, the reduction of KCs population by GdCl_3 _significantly down-regulated the expression of alternative macrophage markers (Arg1, Mrc1) and KCs derived protective cytokine (IL-10) while increasing the expression of pro-inflammatory TNFα and IL-6. We hypothesize that the observed increase in these markers in the liver may reflect the infiltration of macrophages from circulation which have already been pro-inflammatory activated, i.e. in adipose tissue. Much less of an effect was observed in SD administered animals.

It has been demonstrated that KCs depletion either ameliorates [23] or aggravates [25,43] hepatic insulin resistance. The above mentioned observation (the change of the relative abundance of the anti- and pro-inflammatory activated macrophages) may provide the explanation for the different results obtained after acute and chronic GdCl_3 _activation. The acute KCs reduction has different effects on hepatic insulin resistance induced by HF diets with different fat composition. HF diet rich in *trans *FA with high inflammation-stimulating potential evoked severe hepatic insulin resistance and the short-term KCs reduction failed to induce any changes in it, either positive or negative [54]. We explain this by the persisting TNFα expression in the liver as we were not able to completely eliminate classically activated macrophages (TNFα liver expression: pre GdCl_3 _31.3 fold over SD; post GdCl_3 _10 fold over SD). In contrast, HF diet rich in MUFA with low inflammatory potential elicited less pronounced hepatic IR but the IR symptoms which were exacerbated by short-term GdCl_3 _application. Neyrinck [26] using a similar diet (60 cal% as fat, lard) showed that 4 weeks of chronic GdCl_3 _administration resulted in the enhanced whole-body and hepatic insulin sensitivity. Nevertheless, chronically administered GdCl_3 _mice exhibited markedly reduced weight gain on the high fat diet. Improved insulin sensitivity and glucose tolerance in gadolinium treated mice could be due to the decreased weight gain independent of KCs function.

A highly controversial issue is the effect of KCs on hepatic steatosis. KCs have been shown to promote steatosis [43], to reduce steatosis [23] or to have no effect on it [55]. Interestingly, in our experiments, KCs depletion led to a concomitant reduction of liver TAG content. This finding is rather surprising as it has been repeatedly confirmed that ectopic TAG accumulation in the liver is associated with the development of insulin resistance. Nevertheless, studies carried out on a mice model of liver diacylglycerol acyltransferaze-2 overexpression exhibiting severe steatosis but normal hepatic insulin sensitivity [56] showed that steatosis *per se *could be dissociated from the onset if hepatic insulin resistance. According to the currently accepted hypothesis, inert TAG molecule itself probably does not interfere with insulin signaling but the negative effects of steatosis are mediated rather by bioactive intermediates of lipid metabolism like DAG or Lc-AcCoA [57]. This hypothesis is in line with our observation that KCs depletion is associated not only with hepatic insulin resistance but also with significantly accentuated lipolysis and an increased DAG content.

The exact mechanism of the beneficial effect of alternatively activated macrophages on the metabolism of fatty hepatocytes is still a matter of debate. It has been proposed that alternatively activated macrophages may attenuate tissue inflammation by paracrine action which leads to the improved insulin signaling [6,58]. Another hypothesis proposes that alternatively activated macrophages may secrete trophic factors that act in a paracrine or endocrine manner to enhance oxidative metabolism in peripheral tissues [6]. Our data indicates the existence of another mechanism of KCs influence on hepatocytes. In our study, we saw that the reduction of KCs population by GdCl_3 _resulted in a shift towards pro-inflammatory activation with a concomitant decrease of alternative activation. As has been recently shown, pro-inflammatory cytokines, in particular TNF-α, IL-1, IL-2 and IL-6, stimulate autophagy while cytokines produced by alternatively activated KCs, Il-4, IL-10 and IL-13, are inhibitory [59]. We suggest that the reduction of alternatively activated, IL-10 producing macrophages results in the enhancement of autophagy and consequent stimulation of lysosomal lipolysis. This effect could be further potentiated by the increased production of pro-autophagic cytokines like TNFα. In our experiments we observed that the reduction of KCs population resulted in highly significant increase of lysosomal TAG degradation in the steatotic, but not in the normal, liver. The main products of lysosomal lipase action on the TAG molecule are 1,2-sn-DAG and one molecule of fatty acid while the degradation to monoacylglycerol or glycerol and FFA is negligible [60]. 1,2-sn-DAG is an important intracellular signaling molecule and it is the known activator of PKCε [61]. Recently, it has been shown that fat-induced hepatic insulin resistance may result from activation of PKCε and its downstream targets [35,62]. Nevertheless, the source of DAG remained unidentified. The increased DAG content due to the increased flux through TAG synthetic pathway and the following PKCε activation was described in HF diet-fed animals [63]. However, DAG is also an intermediator in the TAG degradation pathway that is accentuated after GdCl_3 _treatment. Our findings suggest that DAG originating from the increased lipolysis could act as a PKCε activator and contributes to the worsening of hepatic insulin sensitivity after KCs reduction.

## Conclusions

We found that a HF diet rich in MUFA triggers the alternative activation program in KCs. In the steatotic liver, a reduction of the KCs population was associated with a decrease of alternative activation and with a shift towards the expression of pro-inflammatory activation markers, with the increased autophagy, elevated lysosomal lipolysis, increased formation of DAG, PKCε activation and marked exacerbation of HF diet-induced hepatic insulin resistance. We propose that in the presence of high MUFA content, the population of alternatively activated resident liver macrophages may mediate the beneficial effects on liver insulin sensitivity and alleviate the metabolic disturbances imposed by a HF diet feeding and steatosis.

## Abbreviations

AUC: Area under the curve; DAG: Diacylglycerol; DHA: Docosahexaenoic acid; EPA: Eicosapentaenoic acid; HF: High-fat diet; KCs: Kupffer cells; Lc-AcCoA: Long-chain acyl coenzyme A; MUFA: Mono-unsaturated fatty acid; NAFLD: Non-alcoholic fatty liver disease; OGTT: Oral glucose tolerance test; PKCε: Protein kinase C epsilon; SFA: Saturated fatty acids; SD: Standard diet; PUFA: Poly-unsaturated fatty acids; TAG: Triac ylglycerols.

## Competing interests

The authors declare that they have no competing interests.

## Authors' contributions

All authors participated in the design, interpretation of the studies and analysis of the data; HD, EP, JZ and ZP conducted the experiments; ZP wrote the manuscript; VS participated in the design of the study and performed the statistical analysis; LK participated in its design and coordination and helped to draft the manuscript. M.C. conceived of the study, analyzed the data and wrote the manuscript. All authors read and approved the final manuscript.

## Supplementary Material

Additional file 1 **Fatty acid composition of lard used for the preparation of the high-fat diet**. The fatty acids were identified using gas chromatography. Briefly, the triacylglycerols were converted into alkalic salts and the released fatty acids were subsequently esterified in alcalic methanol. Methylesters were extracted into heptan and detected by gas chromatography. Click here for file

Additional file 2 **Details of primers used**. Click here for file

Additional file 3 **Antibodies used in Western blot experiments**. Click here for file

Additional file 4 **Weight of the experimental animals**. Yelow fields highlits the days of GdCl3 treatment. EWAT epididymal fat pad. Click here for file

## References

[B1] Rector RS Thyfault JP Wei Y Ibdah JA Non-alcoholic fatty liver disease and the metabolic syndrome: an update World J Gastroenterol 2008 14 185 192 10.3748/wjg.14.185 18186553 PMC2675112

[B2] Hotamisligil GS Shargill NS Spiegelman BM Adipose expression of tumor necrosis factor-alpha: direct role in obesity-linked insulin resistance Science 1993 259 87 91 10.1126/science.7678183 7678183

[B3] Mills CD Kincaid K Alt JM Heilman MJ Hill AM M-1/M-2 macrophages and the Th1/Th2 paradigm J Immunol 2000 164 6166 6173 28923981 10.4049/jimmunol.1701141

[B4] Goerdt S Orfanos CE Other functions, other genes: alternative activation of antigen-presenting cells Immunity 1999 10 137 142 10.1016/S1074-7613(00)80014-X 10072066

[B5] Fessler MB Rudel LL Brown JM Toll-like receptor signaling links dietary fatty acids to the metabolic syndrome Curr Opin Lipidol 2009 20 379 385 10.1097/MOL.0b013e32832fa5c4 19625959 PMC3099529

[B6] Odegaard JI Ricardo-Gonzalez RR Goforth MH Morel CR Subramanian V Mukundan L Red Eagle A Vats D Brombacher F Ferrante AW Chawla A Macrophage-specific PPARgamma controls alternative activation and improves insulin resistance Nature 2007 447 1116 1120 10.1038/nature05894 17515919 PMC2587297

[B7] Rivellese AA De Natale C Lilli S Type of dietary fat and insulin resistance Ann N Y Acad Sci 2002 967 329 335 12079860 10.1111/j.1749-6632.2002.tb04288.x

[B8] Brousseau ME Schaefer EJ Diet and coronary heart disease: clinical trials Curr Atheroscler Rep 2000 2 487 493 10.1007/s11883-000-0048-6 11122783

[B9] Munford RS Hall CL Detoxification of bacterial lipopolysaccharides (endotoxins) by a human neutrophil enzyme Science 1986 234 203 205 10.1126/science.3529396 3529396

[B10] Qureshi N Takayama K Kurtz R Diphosphoryl lipid A obtained from the nontoxic lipopolysaccharide of Rhodopseudomonas sphaeroides is an endotoxin antagonist in mice Infect Immun 1991 59 441 444 1987057 10.1128/iai.59.1.441-444.1991 PMC257761

[B11] Galgani JE Uauy RD Aguirre CA Diaz EO Effect of the dietary fat quality on insulin sensitivity Br J Nutr 2008 100 471 479 10.1017/S0007114508894408 18394213

[B12] Gillingham LG Harris-Janz S Jones PJ Dietary monounsaturated fatty acids are protective against metabolic syndrome and cardiovascular disease risk factors Lipids 2011 46 209 228 10.1007/s11745-010-3524-y 21308420

[B13] Lopez S Bermudez B Abia R Muriana FJ The influence of major dietary fatty acids on insulin secretion and action Curr Opin Lipidol 2010 21 15 20 10.1097/MOL.0b013e3283346d39 19915461

[B14] Bourlier V Bouloumie A Role of macrophage tissue infiltration in obesity and insulin resistance Diabetes Metab 2009 35 251 260 10.1016/j.diabet.2009.05.001 19539513

[B15] Caballero F Fernandez A Matias N Martinez L Fucho R Elena M Caballeria J Morales A Fernandez-Checa JC Garcia-Ruiz C Specific contribution of methionine and choline in nutritional nonalcoholic steatohepatitis: impact on mitochondrial S-adenosyl-L-methionine and glutathione J Biol Chem 2010 285 18528 18536 10.1074/jbc.M109.099333 20395294 PMC2881778

[B16] Sheth K Bankey P The liver as an immune organ Curr Opin Crit Care 2001 7 99 104 10.1097/00075198-200104000-00008 11373518

[B17] Sun Z Wada T Maemura K Uchikura K Hoshino S Diehl AM Klein AS Hepatic allograft-derived Kupffer cells regulate T cell response in rats Liver Transpl 2003 9 489 497 10.1053/jlts.2003.50091 12740792

[B18] Parker GA Picut CA Liver immunobiology Toxicol Pathol 2005 33 52 62 10.1080/01926230590522365 15805056

[B19] Canbay A Feldstein AE Higuchi H Werneburg N Grambihler A Bronk SF Gores GJ Kupffer cell engulfment of apoptotic bodies stimulates death ligand and cytokine expression Hepatology 2003 38 1188 1198 10.1053/jhep.2003.50472 14578857

[B20] Vollmar B Menger MD The hepatic microcirculation: mechanistic contributions and therapeutic targets in liver injury and repair Physiol Rev 2009 89 1269 1339 10.1152/physrev.00027.2008 19789382

[B21] Gordon S Alternative activation of macrophages Nat Rev Immunol 2003 3 23 35 10.1038/nri978 12511873

[B22] Herbert DR Holscher C Mohrs M Arendse B Schwegmann A Radwanska M Leeto M Kirsch R Hall P Mossmann H Claussen B Forster I Brombacher F Alternative macrophage activation is essential for survival during schistosomiasis and downmodulates T helper 1 responses and immunopathology Immunity 2004 20 623 635 10.1016/S1074-7613(04)00107-4 15142530

[B23] Clementi AH Gaudy AM van Rooijen N Pierce RH Mooney RA Loss of Kupffer cells in diet-induced obesity is associated with increased hepatic steatosis, STAT3 signaling, and further decreases in insulin signaling Biochim Biophys Acta 2009 1792 1062 1072 19699298 10.1016/j.bbadis.2009.08.007 PMC2763970

[B24] Rai RM Loffreda S Karp CL Yang SQ Lin HZ Diehl AM Kupffer cell depletion abolishes induction of interleukin-10 and permits sustained overexpression of tumor necrosis factor alpha messenger RNA in the regenerating rat liver Hepatology 1997 25 889 895 10.1002/hep.510250417 9096593

[B25] Lanthier N Molendi-Coste O Horsmans Y van Rooijen N Cani PD Leclercq IA Kupffer cell activation is a causal factor for hepatic insulin resistance Am J Physiol Gastrointest Liver Physiol 2010 298 G107 116 10.1152/ajpgi.00391.2009 19875703

[B26] Neyrinck AM Cani PD Dewulf EM De Backer F Bindels LB Delzenne NM Critical role of Kupffer cells in the management of diet-induced diabetes and obesity Biochem Biophys Res Commun 2009 385 351 356 10.1016/j.bbrc.2009.05.070 19463788

[B27] Committee for the Update of the Guide for the Care and Use of laboratory Animals NIH Guide to the Care and Use of Laboratory Animals 2011 8 National Academies Press: NW Washington

[B28] Brinkhof B Spee B Rothuizen J Penning LC Development and evaluation of canine reference genes for accurate quantification of gene expression Anal Biochem 2006 356 36 43 10.1016/j.ab.2006.06.001 16844072

[B29] Cahova M Dankova H Palenickova E Papackova Z Kazdova L The autophagy-lysosomal pathway is involved in TAG degradation in the liver: the effect of high-sucrose and high-fat diet Folia Biol (Praha) 2010 56 173 182 20974050 10.14712/fb2010056040173

[B30] Belfrage P Vaughan M Simple liquid-liquid partition system for isolation of labeled oleic acid from mixtures with glycerides J Lipid Res 1969 10 341 344 5785006

[B31] Rubinsztein DC Cuervo AM Ravikumar B Sarkar S Korolchuk V Kaushik S Klionsky DJ In search of an "autophagometer" Autophagy 2009 5 585 589 10.4161/auto.5.5.8823 19411822

[B32] Folch J Lees M Sloane Stanley GH A simple method for the isolation and purification of total lipides from animal tissues J Biol Chem 1957 226 497 509 13428781

[B33] Singh R Kaushik S Wang Y Xiang Y Novak I Komatsu M Tanaka K Cuervo AM Czaja MJ Autophagy regulates lipid metabolism Nature 2009 458 1131 1135 10.1038/nature07976 19339967 PMC2676208

[B34] Seglen PO Solheim AE Conversion of dense lysosomes into light lysosomes during hepatocytic autophagy Exp Cell Res 1985 157 550 555 10.1016/0014-4827(85)90141-7 3979450

[B35] Samuel VT Liu ZX Qu X Elder BD Bilz S Befroy D Romanelli AJ Shulman GI Mechanism of hepatic insulin resistance in non-alcoholic fatty liver disease J Biol Chem 2004 279 32345 32353 10.1074/jbc.M313478200 15166226

[B36] Roberts RA Ganey PE Ju C Kamendulis LM Rusyn I Klaunig JE Role of the Kupffer cell in mediating hepatic toxicity and carcinogenesis Toxicol Sci 2007 96 2 15 17122412 10.1093/toxsci/kfl173

[B37] Andres D Sanchez-Reus I Bautista M Cascales M Depletion of Kupffer cell function by gadolinium chloride attenuates thioacetamide-induced hepatotoxicity. Expression of metallothionein and HSP70 Biochem Pharmacol 2003 66 917 926 10.1016/S0006-2952(03)00443-X 12963478

[B38] Muriel P Escobar Y Kupffer cells are responsible for liver cirrhosis induced by carbon tetrachloride J Appl Toxicol 2003 23 103 108 10.1002/jat.892 12666154

[B39] Zhong Z Connor HD Mason RP Qu W Gao W Lemasters JJ Thurman RG Role of Kupffer cells in reperfusion injury in fat-loaded livers from ethanol-treated rats J Pharmacol Exp Ther 1995 275 1512 1517 8531123

[B40] Tsung A Hoffman RA Izuishi K Critchlow ND Nakao A Chan MH Lotze MT Geller DA Billiar TR Hepatic ischemia/reperfusion injury involves functional TLR4 signaling in nonparenchymal cells J Immunol 2005 175 7661 7668 16301676 10.4049/jimmunol.175.11.7661

[B41] Prins HA Meijer C Boelens PG Diks J Holtz R Masson S Daveau M Meijer S Scotte M van Leeuwen PA Kupffer cell-depleted rats have a diminished acute-phase response following major liver resection Shock 2004 21 561 565 10.1097/01.shk.0000126649.96850.36 15167686

[B42] Ellett JD Atkinson C Evans ZP Amani Z Balish E Schmidt MG van Rooijen N Schnellmann RG Chavin KD Murine Kupffer cells are protective in total hepatic ischemia/reperfusion injury with bowel congestion through IL-10 J Immunol 2010 184 5849 5858 10.4049/jimmunol.0902024 20400698 PMC2938026

[B43] Huang W Metlakunta A Dedousis N Zhang P Sipula I Dube JJ Scott DK O'Doherty RM Depletion of liver Kupffer cells prevents the development of diet-induced hepatic steatosis and insulin resistance Diabetes 2010 59 347 357 10.2337/db09-0016 19934001 PMC2809951

[B44] Cintra DE Pauli JR Araujo EP Moraes JC de Souza CT Milanski M Morari J Gambero A Saad MJ Velloso LA Interleukin-10 is a protective factor against diet-induced insulin resistance in liver J Hepatol 2008 48 628 637 10.1016/j.jhep.2007.12.017 18267346

[B45] Xu H Barnes GT Yang Q Tan G Yang D Chou CJ Sole J Nichols A Ross JS Tartaglia LA Chen H Chronic inflammation in fat plays a crucial role in the development of obesity-related insulin resistance J Clin Invest 2003 112 1821 1830 14679177 10.1172/JCI19451 PMC296998

[B46] Sabio G Das M Mora A Zhang Z Jun JY Ko HJ Barrett T Kim JK Davis RJ A stress signaling pathway in adipose tissue regulates hepatic insulin resistance Science 2008 322 1539 1543 10.1126/science.1160794 19056984 PMC2643026

[B47] Shi H Kokoeva MV Inouye K Tzameli I Yin H Flier JS TLR4 links innate immunity and fatty acid-induced insulin resistance J Clin Invest 2006 116 3015 3025 10.1172/JCI28898 17053832 PMC1616196

[B48] Stienstra R Saudale F Duval C Keshtkar S Groener JE van Rooijen N Staels B Kersten S Muller M Kupffer cells promote hepatic steatosis via interleukin-1beta-dependent suppression of peroxisome proliferator-activated receptor alpha activity Hepatology 2010 51 511 522 10.1002/hep.23337 20054868

[B49] Kewalramani G Fink LN Asadi F Klip A Palmitate-activated macrophages confer insulin resistance to muscle cells by a mechanism involving protein kinase C theta and epsilon PLoS One 2011 6 e26947 10.1371/journal.pone.0026947 22046423 PMC3202600

[B50] Bilan PJ Samokhvalov V Koshkina A Schertzer JD Samaan MC Klip A Direct and macrophage-mediated actions of fatty acids causing insulin resistance in muscle cells Arch Physiol Biochem 2009 115 176 190 10.1080/13813450903079314 19671019

[B51] Wen H Gris D Lei Y Jha S Zhang L Huang MT Brickey WJ Ting JP Fatty acid-induced NLRP3-ASC inflammasome activation interferes with insulin signaling Nat Immunol 2011 12 408 415 10.1038/ni.2022 21478880 PMC4090391

[B52] Oliver E McGillicuddy FC Harford KA Reynolds CM Phillips CM Ferguson JF Roche HM Docosahexaenoic acid attenuates macrophage-induced inflammation and improves insulin sensitivity in adipocytes-specific differential effects between LC n-3 PUFA J Nutr Biochem 2011 http://dx.doi.org/10.1016./j.bbr.2011.03.031 10.1016/j.jnutbio.2011.06.014 22137266

[B53] Mullen A Loscher CE Roche HM Anti-inflammatory effects of EPA and DHA are dependent upon time and dose-response elements associated with LPS stimulation in THP-1-derived macrophages J Nutr Biochem 2010 21 444 450 10.1016/j.jnutbio.2009.02.008 19427777

[B54] Papackova Z Palenickova E Dankova H Cahova M Kazdova L The role of tissue macrophages in the development of metabolic syndrome associated dysorders: the effect of high fat diet and the fatty acid composition) Cas Lek Ces 2011 150 185 193

[B55] Lanthier N Molendi-Coste O Cani PD van Rooijen N Horsmans Y Leclercq IA Kupffer cell depletion prevents but has no therapeutic effect on metabolic and inflammatory changes induced by a high-fat diet FASEB J 2011 25 4301 4311 10.1096/fj.11-189472 21873555

[B56] Monetti M Levin MC Watt MJ Sajan MP Marmor S Hubbard BK Stevens RD Bain JR Newgard CB Farese RV Sr Hevener AL Farese RV Jr Dissociation of hepatic steatosis and insulin resistance in mice overexpressing DGAT in the liver Cell Metab 2007 6 69 78 10.1016/j.cmet.2007.05.005 17618857

[B57] Kumashiro N Erion DM Zhang D Kahn M Beddow SA Chu X Still CD Gerhard GS Han X Dziura J Petersen KF Samuel VT Shulman GI Cellular mechanism of insulin resistance in nonalcoholic fatty liver disease Proc Natl Acad Sci USA 2011 108 16381 16385 10.1073/pnas.1113359108 21930939 PMC3182681

[B58] Bouhlel MA Derudas B Rigamonti E Dievart R Brozek J Haulon S Zawadzki C Jude B Torpier G Marx N Staels B Chinetti-Gbaguidi G PPARgamma activation primes human monocytes into alternative M2 macrophages with anti-inflammatory properties Cell Metab 2007 6 137 143 10.1016/j.cmet.2007.06.010 17681149

[B59] Harris J Autophagy and cytokines Cytokine 2011 56 140 144 10.1016/j.cyto.2011.08.022 21889357

[B60] Hayase K Tappel AL Specificity and other properties of lysosomal lipase of rat liver J Biol Chem 1970 245 169 175 5413966

[B61] Nishizuka Y Protein kinase C and lipid signaling for sustained cellular responses FASEB J 1995 9 484 496 7737456

[B62] Samuel VT Liu ZX Wang A Beddow SA Geisler JG Kahn M Zhang XM Monia BP Bhanot S Shulman GI Inhibition of protein kinase Cepsilon prevents hepatic insulin resistance in nonalcoholic fatty liver disease J Clin Invest 2007 117 739 745 10.1172/JCI30400 17318260 PMC1797607

[B63] Schmitz-Peiffer C Browne CL Oakes ND Watkinson A Chisholm DJ Kraegen EW Biden TJ Alterations in the expression and cellular localization of protein kinase C isozymes epsilon and theta are associated with insulin resistance in skeletal muscle of the high-fat-fed rat Diabetes 1997 46 169 178 10.2337/diabetes.46.2.169 9000691

