# Supplementary material for: Kupffer cells ameliorate hepatic insulin resistance induced by high-fat diet rich in monounsaturated fatty acids: the evidence for the involvement of alternatively activated macrophages
Source: Nutr Metab (Lond). 2012 Mar 22;9:22. doi: 10.1186/1743-7075-9-22 (PMC3348013; doi:10.1186/1743-7075-9-22)
Supplement: Additional file 1 — Fatty acid composition of lard used for the preparation of the high-fat diet. The fatty acids were identified using gas chromatography. Briefly, the triacylglycerols were converted into alkalic salts and the released fatty acids were subsequently esterified in alcalic methanol. Methylesters were extracted into heptan and detected by gas chromatography. [file 1743-7075-9-22-S1.DOC]

**Supplement 1**

**Fatty acid composition of lard used for the preparation of the high-fat diet.**

|  |  | **%** |
| --- | --- | --- |
| myristic acid | **C14** | **4** |
| palmitic acid | **C16** | **20** |
| palmitooleic acid | **C16:1cis** | **4** |
| heptadekanoic | **C17** | **0.5** |
| stearic acid | **C18** | **12** |
| oleic acid | **C 18:1** | **51** |
| linoleic acid | **C18:2cis** | **8** |
| linolenic acid | **C18:3cis** | **0.5** |

The fatty acids were identified using gas chromatography. Briefly, the triacylglycerols were converted into alkalic salts and the released fatty acids were subsequently esterified in alcalic methanol. Methylesters were extracted into heptan and detected by gas chromatography
