# Supplementary material for: Kupffer cells ameliorate hepatic insulin resistance induced by high-fat diet rich in monounsaturated fatty acids: the evidence for the involvement of alternatively activated macrophages
Source: Nutr Metab (Lond). 2012 Mar 22;9:22. doi: 10.1186/1743-7075-9-22 (PMC3348013; doi:10.1186/1743-7075-9-22)
Supplement: Additional file 2 — Details of primers used. [file 1743-7075-9-22-S2.DOC]

**Suplement 2**

**Details of primers used**

| Gene | GeneID | SENSE PRIMER  (5´ - 3´) | ANTISENSE PRIMER  (5´ - 3´) |
| --- | --- | --- | --- |
| CD68 | NM_001031638.1 | CAAGCAGCACAGTGGACATTC | GGCAGCAAGAGAGATTGGTC |
| Emr | NM_001007557.1 | CAACCGCCAGGTACGAGATG | TGCCGCCAACTAACGATACC |
| IL-6 | NM_012589.1 | GAGAAAAGAGTTGTGCAATGG | ACGGAACTCCAGAAGACCAG |
| IL-1β | NM_031512.1 | GCCAACAAGTGGTATTCTCC | GTTTGGGATCCACACTCTCC |
| Arg-1 | NM_017134.1 | CTGCTGGGAAGGAAGAAAAG | ACTGCCGTGTTCACAGTACG |
| Mrc1 | NM_001106123.1 | CAACCAAAGCTGACCAAAGG | AGGTGGCCTCTTGAGGTATG |
| IL-10 | [NM_012854.2](http://www.ncbi.nlm.nih.gov/nuccore/NM_012854.2) | CCCTCTGGATACAGCTGCG | GCTCCACTGCCTTGCTTTTATT |
| Ubc | NM_017314.1 | CACCAAGAAGGTCAAACAGG | GAACTTTATTCAAAGTGCAATGAAAC |
