# Supplementary material for: Kupffer cells ameliorate hepatic insulin resistance induced by high-fat diet rich in monounsaturated fatty acids: the evidence for the involvement of alternatively activated macrophages
Source: Nutr Metab (Lond). 2012 Mar 22;9:22. doi: 10.1186/1743-7075-9-22 (PMC3348013; doi:10.1186/1743-7075-9-22)
Supplement: Additional file 3 — Antibodies used in Western blot experiments. [file 1743-7075-9-22-S3.DOCX]

| Antibody | Phosphorylation  site | Ref. No. | Company | Dilution |
| --- | --- | --- | --- | --- |
| Phospho-Akt | Ser473 | #9271 | Cell Signaling | 1:1000 |
| Akt |  | #9272 | Cell Signaling | 1:700 |
| Anti-Insulin Receptor | Tyr1158 | ab78355 | Abcam, Camebridge, UK | 1:750 |
| Anti-Insulin Receptor |  | ab5500 | Abcam, Camebridge, UK | 1:1000 |
| Mouse monoclonal (9G7F12) to lysosomal acid lipase |  | ab36597 | Abcam, Camebridge, UK | 1:1000 |
| rabbit polyclonal to LC3A/B |  | ab58610 | Abcam, Camebridge, UK | 1:1000 |
| β-actin |  | #4967 | Cell Signaling | 1:3000 |

**Supplement 3**

**Antibodies used in Western blot experiments**
